# Medicines not recommended for inclusion in the who essential medicines list: a retrospective observational study

**DOI:** 10.3389/fmed.2025.1517020

**Published:** 2025-03-17

**Authors:** Enrico Costa, Vittorio Del Grosso, Bernadette Cappello, Armando A. Genazzani, Benedikt Huttner, Hubert G. M. Leufkens, Nicola Magrini, Francesco Nonino, Veronika J. Wirtz, Hendrika A. van den Ham, Lorenzo Moja

**Affiliations:** ^1^Utrecht World Health Organization Collaborating Centre for Pharmaceutical Policy and Regulation, Division of Pharmacoepidemiology and Clinical Pharmacology, Utrecht University, Utrecht, Netherlands; ^2^Department of Pharmaceutical Sciences, University of Eastern Piedmont, Novara, Italy; ^3^Essential Medicines Team, Department of Health Products Policy and Standards, Access to Medicines and Health Products Division, World Health Organization, Geneva, Switzerland; ^4^Department of Drug Science and Technology, University of Turin, Turin, Italy; ^5^Department of Surveillance, Prevention and Control, Antimicrobial Resistance Division, World Health Organization, Geneva, Switzerland; ^6^Emeritus Professor, Regulatory Science and Pharmaceutical Policy, Utrecht University, Utrecht, Netherlands; ^7^WHO Collaborating Centre for Evidence-Based Research Synthesis and Guideline Development, Emilia-Romagna, Italy; ^8^WHO Collaborating Centre in Pharmaceutical Policy, Department of Global Health, Boston University, Boston, MA, United States

**Keywords:** essential medicines, health technology assesement (HTA), inappropriate healthcare, negative recommendation list, medicine policies

## Abstract

**Background:**

The WHO Model List of Essential Medicines (EML) includes those medicines that offer the best health payback for individuals and health systems. It serves as a guide for countries to develop and update national EMLs. The implementation of essential medicines policies is therefore mostly oriented to medicines on the EML. However, medicines evaluated and not recommended for inclusion in the EML also have relevant implications for development of efficient medicine policies. This study analyzed the characteristics, frequencies, and reasons for applications for medicines proposed for inclusion in the WHO EML not being recommended.

**Methods:**

Assessment of the recommendations for all medicines proposed for inclusion in the WHO EML in reports of the Expert Committee on Selection and Use of Essential Medicines in the WHO Technical Reports Series from 2002 to 2023. We collected key information from EML applications including active substance, therapeutic indication, orphan status, applicant, and reasons for negative recommendations. Logistic univariate and multivariate regression analyses assessed predictive characteristics for applications with negative recommendations.

**Results:**

A total of 359 applications for addition of new medicines to the EML were submitted: 211 (58.8%) received a positive recommendation. Among the 148 (41.2%) applications with a negative recommendation, the most prevalent reasons for not recommending were quality of clinical evidence (62.1%) and economic criteria (33.1%). Concerns about capacity to implement the new medicines in health care systems or requiring specialized expertise increased over time. Applications submitted by pharmaceutical companies, individuals not affiliated with scientific societies or non-governmental organizations, and academia were more prone to receiving a negative recommendation.

**Discussion:**

An appreciable proportion of applications for addition of new medicines to the EML are not recommended. Over time, low or limited quality of clinical evidence was a consistent explanatory reason leading to non-recommending. Economic considerations and feasibility are emerging justifications for non-recommending.

## Background

In 1977, the World Health Organization (WHO) published the first Model List of Essential Medicines (EML), a list comprising those medications considered “of utmost importance, basic, indispensable and necessary for the health and needs of the population” (1). The EML serves as guidance supporting countries in developing and updating their own national EMLs, and for international organizations (e.g., UNICEF) to prioritize the procurement and supply of medicines (2, 3).

While the items recommended in the EML are updated every 2 years, the structure of the EML has remained substantially unchanged over time. Medicines intended for the treatment of priority conditions in a basic healthcare system are listed in the ‘core list,’ while those requiring specialized facilities and/or expertise for their use are listed in the ‘complementary list.’ However, the EML has undergone several conceptual and operational changes over the past few decades (Box 1) (4).

BOX 1 **Milestones in the evolution of the WHO Model List of Essential Medicines List.**

**1977** - First Model List of Essential Drugs recommending 208 medications (WHO TRS No. 615) (1).

**1983** - Introduction of the square box symbol to indicate therapeutic equivalence of medicines within a pharmacological class of therapeutic group (WHO TRS No. 685) (5, 6).

**2001** - Revised procedure for updating the WHO’s Model List of Essential Drugs. The name changed from Essential Drugs to Essential Medicines (WHO. 2001. EB109/8) (7).

**2002** - Several patented antiretrovirals for the treatment of HIV were recommended (WHO TRS o.914) (8).

**2007** - First Model List of Essential Medicines for Children (EMLc) (WHO TRS No. 950) (9).

**2015** - Comprehensive review of essential medicines for cancer, leading to the recommendations of first monoclonal antibodies (WHO TRS No.994) (10, 11).

**2020** - Launch of the electronic EML (eEML), an easy-to-use digital version of the EML (12).

On a biennial basis, following an open call, public institutions, scientific and medical organizations, pharmaceutical companies and individuals, can submit applications for the addition or removal of new medicines on the WHO EML, as well as propose changes to existing listed medicines (e.g., new formulations and indications) (2). Applications are reviewed by an independent, international and multidisciplinary WHO Expert Committee that provides non-binding recommendations to the WHO Director General. In 2001, in response to growing methodological concerns, the process for selecting essential medicines became more structured, shifting from an opinion-based to an evidence-based approach, grounded on dimensions such as public health relevance, evidence of efficacy and safety, and cost-effectiveness (7). The absolute cost of a medicine was no longer considered a barrier to being recommended in the EML provided the other selection criteria were met. It was recognized that cost was a factor that could be potentially modified through political engagement once the medicine is recommended for EML listing (13, 14).

The EML is known as a positive list. It is desirable that the implementation of the list at the country level is focused on medicines that have received a positive recommendation from the WHO. However, medicines that have been evaluated and received a negative recommendation have been subject to the same amount of scrutiny as those that received a positive recommendation. Therefore, medicines with negative recommendations also have a high informative value for healthcare decision-making (15). They may lack efficacy, safety, equity, feasibility, cost-effectiveness, or be associated with other limitations. If information on medicines with negative recommendations is suppressed, key medicine limitations can be unnoticed by other panels and national health authorities (16, 17).

We analyzed the characteristics, frequencies, and reasons for negative medicines recommendations and identified potentially relevant decision patterns.

## Methods

### Design and data extraction

In this retrospective observational study, we analyzed the accounts of the EML Expert Committee recommendations published in the WHO Technical Report Series (TRS) from 2002 (when the revised EML selection criteria were introduced) to 2023. These reports record the full recommendations made by the Expert Committee for each EML update for all applications evaluated. In line with the WHO EML process, we considered each EML application as the unit of the analysis.

We selected only applications for the inclusion of new medicines. We excluded applications requesting the addition of a new formulation, dosage form, or strength of a medicine. Each application was dichotomously coded as recommended (i.e., positive recommendation) or not recommended (i.e., negative recommendation), irrespective of being proposed for the core or complementary list, the EML or EMLc. Single applications proposing multiple different medicines were categorized as recommended when at least one medicine was recommended.

For each application, we retrieved data on active substances, therapeutic indications, applicant, and orphan designation granted by the US Food and Drug Administration (FDA) or the European Medicines Agency (EMA) (18, 19). We categorized applicants into the following groups: WHO (e.g., technical departments or units within WHO); WHO collaborating centres; academia; healthcare institutions; non-governmental organizations (NGOs, e.g., patient organizations, scientific societies, etc.); individuals; and pharmaceutical companies.

We collected reported reasons for negative recommendations and categorized reasons into eight main domains, which are closely aligned to WHO EML assessment dimensions (14, 20): (1) disease, the target condition is not considered a public health priority or not considered in WHO guidelines; (2) efficacy, concerns over limited benefits, preference for better alternative in class for benefit; (3) safety, concerns about toxicity, pharmacodynamics or pharmacokinetics, harmful interactions (e.g., drug–drug, drug-food); (4) quality of clinical evidence, cumulation of clinical data was considered insufficient or immature at the time of submission, or data provided by applicants considered not complete; (5) supply, limitations related to limited availability of the product, or to production or supply chain issues, or; (6) regulatory, medicines not approved by stringent regulatory authorities, or concerns on meeting regulatory compliance at global level; (7) feasibility, risks of inappropriate use, concerns on dosing regimens, or limitations of compliance/adherence, limited transferability of the intervention in low-resourced settings due to the need for high-level expertise and sophisticated facilities; (8) economic criteria, when information about cost is lacking, or the proposed medicines is not considered cost effective (e.g., increment in price not proportional to increment in net benefit or better alternatives in class for price are available). For applications with a negative recommendation for multiple reasons, we reported all reasons mentioned in the TRS categorizing them according to our classification. We could not rank reasons leading to negative recommendations as the Technical Reports do not discriminate between primary and secondary reasons.

Two researchers (VDG, EC) independently collected and entered data in an MS Excel database comparing data entry for inconsistency. Persisting doubts were solved by a third author (LM).

### Data analysis

Numbers and proportions of applications with negative recommendations over applications with positive recommendations are presented for each update of the EML as well as reasons for negative recommendations.

Quantitative and qualitative differences between positive and negative recommendations for applications were investigated by stratification of the following variables: type of list (EML or EMLc, core and/or complementary), therapeutic group (communicable or non-communicable diseases), active substance (chemical or biological), orphan designation, and type of applicant. Univariate and multivariate odds ratios (OR), including 95% confidence intervals (95%CI) were calculated using logistic regression to assess the predictive value of these variables. Analyses were performed using SPSS software version 28.0.1.1 (21).

## Results

From 2002 to 2023, a total of 359 applications were submitted to the EML, with 137 (38.2%) applications involving the EMLc. Most applications regarded the core list (216/359; 60.2%), non-communicable diseases (225/359; 62.7%), chemical molecules (308/359; 85.8%), and non-orphan medicines (291/359; 81.1%). Among applications for non-communicable diseases, a wide range of therapeutic areas were represented, with cancer accounting for the largest proportion (64/225; 28.0%). NGOs were responsible for submitting 117/359 (32.6%) applications, with a marked increase in the last decade. WHO consistently submitted applications over time, being responsible for 89/359 (24.8%) applications (Table 1).

**Table 1:** Characteristics of applications submitted to the WHO Essential Medicines List between 2002 and 2023.

	Total applications submitted *N* = 359	
List	*N*	%
EML	222	61.8%
EML AND EMLc	106	29.5%
EMLc	31	8.6%
Listing		
Core	216	60.2%
Complementary	134	37.3%
Core AND Complementary	9	2.5%
Therapeutic group		
Communicable	134	37.3%
Noncommunicable	225	62.7%
*Cancer*	*64*	*17.8%*
*Neurological/mental health*	*33*	*9.2%*
*Blood/Cardiovascular*	*30*	*8.4%*
*Immuno-inflammatory*	*23*	*6.4%*
*Metabolism*	*22*	*6.1%*
*Others*	*53*	*14.8%*
*Active substance*		
Chemical	308	85.8%
Biological	51	14.2%
*Orphan designation*		
No	291	81.1%
Yes	68	18.9%
*Applicant*		
Non-profit organization	117	32.6%
WHO	89	24.8%
Pharmaceutical company	64	17.8%
Academia	55	15.3%
Healthcare institution	25	7.0%
WHO Collaborating Center	21	5.8%
Individual	20	5.6%
NA	*6*	1.7%

### Applications with a negative recommendation

**Figure 1:**
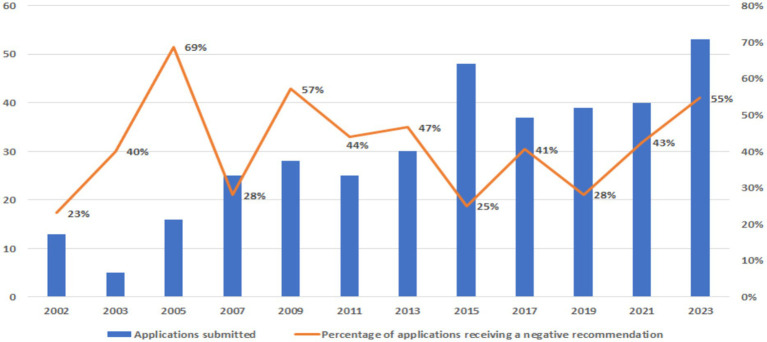
Applications submitted to the WHO EML between 2002 and 2023 with negative recommendations.

Overall, 148/359 (41.2%) applications received a negative recommendation. While the number of applications increased over time, the percentage of applications with negative recommendations showed fluctuations, ranging from around 25% (in 2002, 2007, and 2015) to 69% in 2005 (Figure 1).

### Reasons for negative recommendations

**Figure 2:**
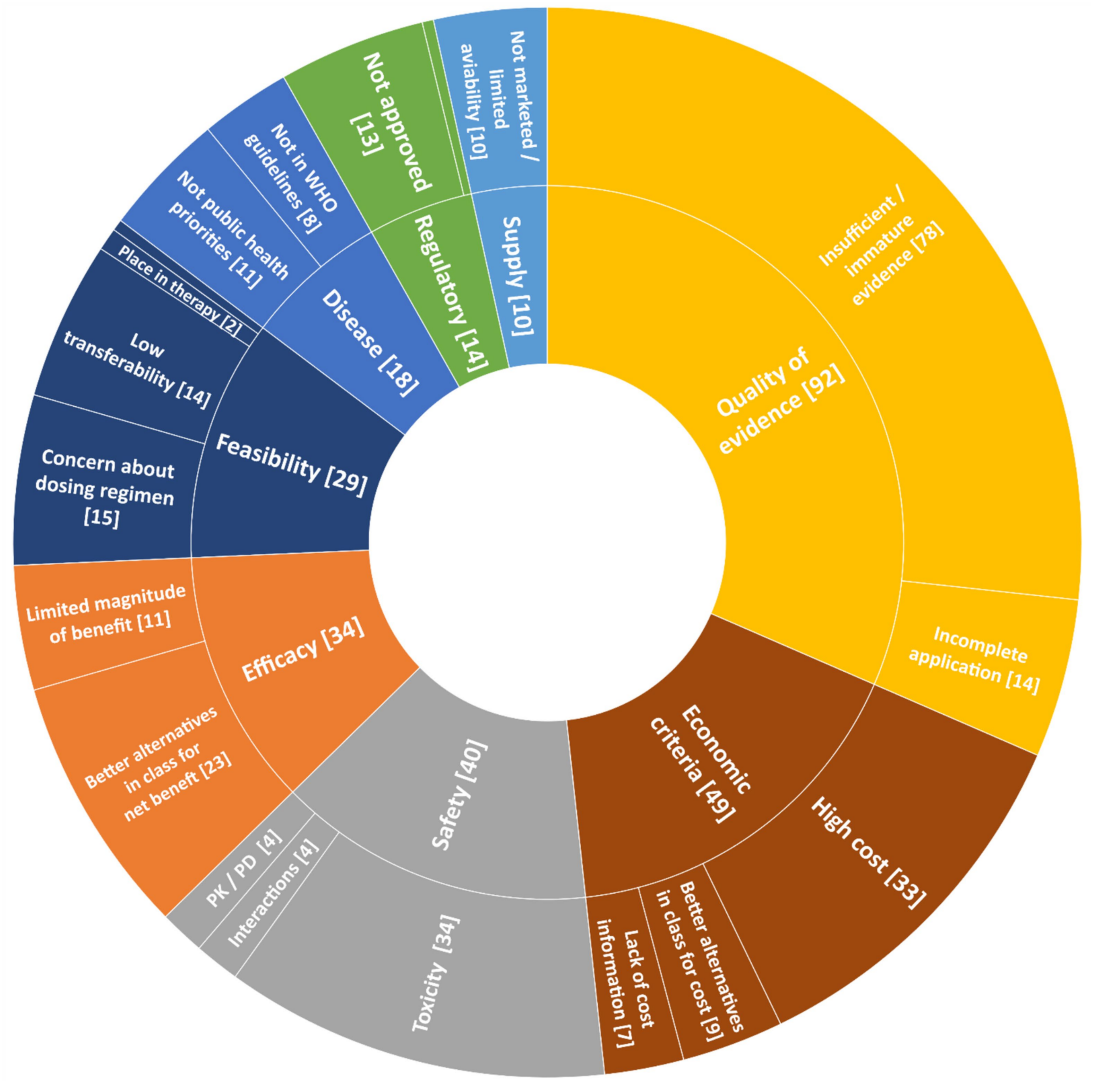
Reasons for negative recommendations of WHO EML applications between 2002 and 2023. Symbols: * Inappropriate use [1]; ° Manufacturing quality [1]. PK, pharmacokinetics; PD, pharmacodynamics.

Reasons for negative recommendations between 2002 and 2023 are reported in Figure 2. The most frequent reasons were quality of clinical evidence (92 cases, 62.1%), economic criteria (49 cases, 33.1%), and safety (40 cases, 27.0%). In 58 out of 148 (39.2%) applications, only one reason was provided in support of the negative recommendation, while for the remaining 90 (60.8%) applications with a negative recommendation, multiple reasons were presented.

**Figure 3:**
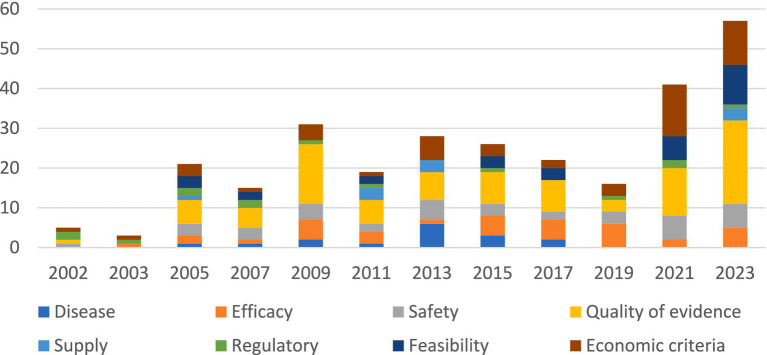
Reasons for negative recommendations of WHO EML applications between 2002 and 2023 per update year.

In Figure 3 reasons for negative recommendations are stratified by EML update year. In the 2021 and 2023 EML updates, there was an increase in the percentage of the quality of clinical evidence, economic criteria, and feasibility as a reason for non-inclusion.

Concerns over the quality of evidence were the most prevalent reason for negative recommendations across all therapeutic groups and applicants, reaching the highest proportion for the pharmaceutical industry (27/32 rejections, 84.4%). Unfavorable cost-effective data was the main justification for rejection (42/49 rejections, 85.7%). Economic criteria mostly concerned cancer medicines (20/49 rejections, 40.1%) followed by immune-inflammatory modulators (9/49 rejections, 18.4%). No applications were rejected only for high prices.

The figure shows the cumulative number of reasons for rejections per year.

### Predictors for negative recommendations

In univariate analysis, applications receiving negative recommendations were significantly associated with biological molecules (OR: 1.91; CI95% 1.05–3.46), type of applicant—academia (OR: 2.98; 95%CI 1.31–6.80), pharmaceutical companies (OR: 3.69; 95%CI 1.81–7.52) and individuals (OR: 4.57; 95%CI 1.59–13.16). In multivariate analysis, applications receiving negative recommendations were significantly associated with potential listing in the complementary list (OR: 1.93; 95%CI 1.11–3.36) and type of applicant - pharmaceutical companies (OR: 4.00; 95%CI 1.88–8.55) or individuals (OR: 3.37; 95%CI 1.02–11.11). Applications for medicines for non-communicable diseases (OR: 0.63; 95%CI 0.40–0.98) were associated with decreased odds of a negative recommendation in univariate analysis. Applications for medicines with orphan status were associated with decreased odds of a negative recommendation in both univariate (OR: 0.49; 95%CI 0.27–0.87) and multivariate analyses (OR: 0.28; 95%CI 0.14–0.55) (Table 2).

**Table 2:** Association of characteristics of EML applications leading to negative recommendations between 2002 and 2023 (univariate and multivariate logistic regression).

	Applications submitted (*N* = 359)	Negative recommendations (*N* = 148)	Negative/total	Univariate OR (95% CI)	Multivariate OR (95% CI)
List					
EML	222	95	0.43	1.00	1.00
EML&EMLc	106	36	0.34	0.69 (0.42–1.11)	0.62 (0.35–1.04)
EMLc	31	17	0.55	1.62 (0.76–3.46)	1.88 (0.81–4.35)
Listing					
Core	216	81	0.38	1.00	1.00
Complementary	134	62	0.46	1.43 (0.93–2.22)	**1.93 (1.11–3.36)**
Core & Complementary	9	5	0.56	2.08 (0.54–8.00)	3.28 (0.75–14.29)
Therapeutic group					
Communicable	134	46	0.34	1.00	1.00
Noncommunicable	225	102	0. < 45	**0.63 (0.40–0.98)**	0.70 (0.37–1.30)
Active substance					
Chemical	308	120	0.39	1.00	1.00
Biological	51	28	0.55	**1.91 (1.05–3.46)**	1.94 (0.94–3.98)
Orphan designation					
No	291	129	0.44	1.00	1.00
Yes	68	19	0.28	**0.49 (0.27–0.87)**	**0.28 (0.14–0.55)**
Single applicant					
WHO	77	21	0.27	1.00	1.00
Non-profit organization	99	37	0.37	1.59 (0.83–3.04)	1.13 (0.52–2.45)
Pharmaceutical company	62	36	0.58	**3.69 (1.81–7.52)**	**4.00 (1.88–8.55)**
Academia	36	19	0.53	**2.98 (1.31–6.80)**	2.31 (0.89–6.06)
Healthcare institution	10	3	0.30	1.14 (0.27–4.83)	0.90 (0.19–4.35)
WHO Collaborating Center	17	4	0.24	0.82 (0.24–2.80)	0.54 (0.14–2.16)
Individual	19	12	0.63	**4.57 (1.59–13.16)**	**3.37 (1.02–11.11)**
Multiple applicants	33	14	0.42	1.96 (0.84–4.61)	2.05 (0.78–5.38)
NA	6	2	0.33	1.33 (0.23–7.81)	0.81 (0.12–5.41)

Bold values indicates statistical significance.

## Discussion

This study analyzed the phenomenon of applications for the inclusion of new medicines in the WHO EML or EMLc which received negative recommendations. A negative recommendation for candidate essential medicines is a frequent event, seen in about 40% of all applications, and characterized by important fluctuations over time. If reasons underlying rejections are taken at face value, the EML process could be considered resistant to promoting medicines not supported by a firm evidence base. It is possible that the clinical evidence supporting candidate essential medicines matures with time, leading to positive recommendations to include medicines that had previously received a negative recommendation. Possibly, the inconsistency in maturation of clinical evidence over time may explain the above-mentioned fluctuating rejection trends observed in time (22). As the membership of the EML Expert Committee changes at least partially with each update, the fluctuation might also reflect different approaches among Committees. However, in 2024 a study investigating the composition and characteristics of the stakeholders that prioritize essential medicines did not identify evidence of change in professional expertise, although over recent years an increased proportion of the members were from low-income and middle-income countries (23).

Applications submitted by pharmaceutical companies, academia, and individuals not affiliated with scientific societies or NGOs were more likely to receive a negative recommendation, as well as medicines requiring specialized facilities and/or expertise (i.e., medicines proposed for the complementary list), and biological molecules. Negative recommendations observed for pharmaceutical companies could be explained by the desire of big corporations to seek the status of essential medicines for new molecules so to rapidly scale up the global market. However, medicines sponsored by pharmaceutical companies are also often characterized by high costs and low feasibility, as well as use of premature evidence supporting the applications, undermining the request.

Applications for medicines with an orphan status were less likely to receive a negative recommendation when compared to applications for non-orphan medicines. It should be noted that only a small proportion of medicines with an approved orphan status were evaluated for the EML in the last decades (24). Applicants may have prioritized for submission those orphan medicines meeting the EML criteria (24). Another explanation is that some medicines with orphan status do not target rare diseases as such, but rather diseases that are rare in some parts of the world but prevalent in others, e.g., hemoglobinopathies or malaria (25, 26). This could have biased the genuine comparison between orphan and non-orphan medicines.

The main reason for negative recommendations for applications emerged as concerns about the quality of clinical evidence, which accounted for around two-thirds of such outcomes. In most cases, the clinical evidence was deemed too immature or insufficient to support a positive recommendation. Although assessing the level or certainty of evidence of applications submitted to WHO was out of the scope of the present analysis, our findings suggested the central role of the quality of evidence presented in the application. Other studies assessing the application quality (e.g., how diligent the authors follow the application instructions and the level and quality of evidence provided) found that it varies and likely hampers decision making of the EML Committee (13, 27). In 2024 the WHO published expert recommendations to implement mechanisms to ensure quality of the applications submitted and considered by the EML Committee (17).

One-third of negative recommendations concerned economic criteria. Our findings suggest a prudent approach in recommending highly priced medicines often used in secondary care institutions, in which limited feasibility in low-resourced settings could hamper medication safety and efficient use. The implications of highly priced medicines in the context of EML decisions are fiercely debated (15, 28). Another reason behind the negative recommendations could be the need to slow down the uptake of new medicines in order to give Member States sufficient time to implement policies increasing access to medicines already recommended. The reiterated negative recommendations for insulin long-acting analogs or polypill for preventing cardiovascular events could also be ascribed to this reason (29, 30).

This study has some limitations. First, we could have underestimated the rate of not recommended medicines, as we considered the whole application as the unit of the analysis. For applications covering multiple medicines, for which only some of the proposed medicines received a positive recommendation, our findings did not include the medicines that were not recommended. We did not assess whether a medicine that had received a negative recommendation had subsequently been recommended following a new application. Resubmission of the same medicine or class of medicines over time is not uncommon. It is possible that medicines that are consistently rejected differ in characteristics from those that are first rejected and then recommended. Lastly, the lack of a formal ranking in the reasons for negative recommendations in the EML Technical Reports prevents the identification of main reasons from ancillary ones.

## Conclusion

Most of the attention on essential medicines for universal health care programs is focused on medicines that are included in the WHO EML. This limits credit to the scrupulous work done in evaluating medicines that have been determined not to meet the criteria of essential and thus are not included on the WHO EML. Information on WHO decision-making has progressively improved, providing comprehensive evidence summaries and reasons behind the EML Expert Committees’ recommendations (31). This information is publicly and freely available in WHO Technical Report Series reports and online in the electronic EML (eEML) database (32). The careful assessment as global level provides an important input to the selection process of essential medicines at national level. At present, negative recommendations are not valued, as they are difficult to access (i.e., they have to be searched manually). Although EML recommendations are not binding on countries, when defining national therapeutic priorities, not only should clinicians and policymakers consider the medicines on the EML, but also those medicines that have been evaluated for inclusion and have not been recommended. The EML process could be improved by making available an updated and easily-accessible list reporting the negative recommendations.

To improve the development and evaluation of applications, better guidance should be provided to applicants regarding minimum quality standards for the evidence base. This should include valid methodological tools to assess and summarize economic criteria. The road to universal health coverage will be facilitated by recognizing what does not offer the best clinical and financial payback for health care systems.

## Data Availability

The datasets presented in this article are not readily available because the data that support the findings of this study are available on request from the corresponding author, Dr Enrico Costa. Data are available with the permission of WHO EML Secretariat. Requests to access the datasets should be directed to Enrico Costa, e.costa@uu.nl.
